# Preparation and Characterization of Dihydromyricetin-Loaded Poly(vinyl alcohol)/Gelatin/Zein Composite Electroblowing Nanofibers

**DOI:** 10.3390/foods15142441

**Published:** 2026-07-09

**Authors:** Hui Xiang, Qin Li, Longchen Shang, Xiujuan Chen, Lingli Deng, Yexing Tao

**Affiliations:** College of Biological and Food Engineering, Hubei Minzu University, Enshi 445000, China; 202430415@hbmzu.edu.cn (H.X.);

**Keywords:** food packaging, nanofibers, DMY, antioxidant, antibacterial

## Abstract

In this study, composite nanofibrous membranes composed of poly(vinyl alcohol) (PVA), gelatin, and zein loaded with different contents of dihydromyricetin (DMY) were fabricated via electroblowing spinning (EBS). The effects of DMY content on the microstructure, physicochemical properties, mechanical strength, and functional performance of the membranes were evaluated. Scanning electron microscopy (SEM) analysis showed that the average fiber diameter increased from 174 ± 29 nm to 221 ± 35 nm with increasing DMY content, followed by a slight decrease at higher loading levels, indicating that DMY incorporation influences fiber morphology. Fourier transform infrared spectroscopy (FTIR) results suggested the presence of hydrogen bonding interactions between DMY and the polymer matrix. X-ray diffraction (XRD) and differential scanning calorimetry (DSC) results indicated changes in the physical state of DMY within the nanofibrous system as the loading content increased. All samples exhibited a typical two-stage release behavior, and the highest cumulative release (nearly 55%) was observed at a DMY loading of 22.5%, while further increasing the loading reduced the release efficiency to approximately 45%. The release profiles were well described by a first-order kinetic model. The composite membranes exhibited improved surface hydrophilicity, appropriate water vapor permeability, antioxidant activity, and antibacterial activity against *Escherichia coli* (*E. coli*) and *Staphylococcus aureus* (*S. aureus*). This study demonstrates the successful fabrication of DMY-loaded PVA/gelatin/zein nanofibrous membranes and provides preliminary insights into their structure–property–function relationships, release behavior, antioxidant activity, and antibacterial activity against representative bacteria, although further application-oriented validation is still required.

## 1. Introduction

Food packaging is essential for protecting food quality and safety [1]. While petroleum-based polymers dominate the market due to their cost-effectiveness and mechanical strength, their environmental persistence and lack of active protection against microbial and oxidative spoilage pose significant challenges [2]. Consequently, research has shifted toward eco-friendly, bioactive alternatives. Electrospinning has emerged as a key technique, producing nanofibrous membranes with high porosity and surface area, ideal for encapsulating active compounds [3,4,5]. Electroblowing spinning (EBS) further enhances this process by combining airflow with electrostatic fields, improving production efficiency and structural control. EBS has attracted considerable attention as an efficient technique for fabricating nanofibrous materials with high productivity and tunable microstructures. Previous studies have demonstrated that EBS can be successfully applied to a variety of polymer systems, producing nanofibrous membranes with uniform morphology, good flexibility, and continuous fibrous structures suitable for functional packaging applications [6,7,8]. In addition, EBS-fabricated biopolymer-based materials, such as cellulose/chitosan composites, have shown enhanced antibacterial activity, hydrophobicity, and mechanical flexibility, highlighting their potential for sustainable food packaging applications [9]. Compared with conventional casting and electrospinning methods, EBS also offers advantages in terms of material dispersion, membrane porosity, and production efficiency, facilitating the scalable fabrication of multifunctional packaging materials [10]. These findings collectively establish EBS as a promising platform for developing high-performance and environmentally friendly food packaging systems.

In recent research, biopolymer-based electrospun nanofibers have shown broad application prospects in food-packaging research due to their biodegradability, film-forming properties, and capacity to incorporate bioactive compounds. Poly(vinyl alcohol) (PVA) has attracted considerable attention among such materials because of its excellent water solubility, film-forming ability, high mechanical strength, and biocompatibility [11]. The abundant hydroxyl groups in PVA molecules confer good hydrophilicity, which is beneficial for enhancing biological interactions, but also lead to poor structural stability in aqueous media. To improve the performance, Carla et al. [12] blended PVA with chitosan for electrospinning, which not only optimized the fiber morphology but also increased the tensile strength of the composite fibers to 9.98 MPa. Similarly, Melbi et al. [13] introduced cellulose to prepare cellulose/PVA nanofibrous membranes, resulting in a 28.2% increase in tensile strength and a 1.6% improvement in thermal stability. Gelatin and zein offer complementary functional benefits: gelatin provides excellent electrospinnability [14], whereas the hydrophobic nature of zein significantly enhances water resistance and thermal stability [15]. Research indicates that gelatin/zein composites achieve superior flexibility and deformability compared to their individual components [16,17,18]. Given that single-polymer systems rarely satisfy the complex mechanical and barrier requirements of food packaging, “blending modification” has become a pivotal strategy for performance optimization [19,20]. While binary systems are well-documented, research on PVA/gelatin/zein ternary blends remains limited. This study leverages the synergistic effects of these three polymers to develop multifunctional nanofibers with a balanced performance profile tailored for advanced packaging applications.

Dihydromyricetin (DMY), a natural flavonoid with potent antioxidant and antibacterial activities, is a highly promising bioactive ingredient for food preservation [21,22]. Despite its potential, most current research remains clinically oriented. For instance, Guo et al. [23] demonstrated that pectin/chitosan nanofibers loaded with 15% DMY achieved a 99.5% wound healing rate in rats by day 9, characterized by organized collagen formation and minimal inflammation. Similarly, Wang et al. [24] found that a multilayer DMY-nanofiber system reduced wound healing time by 32% in diabetic mice while significantly increasing collagen deposition (58%) and angiogenesis (60%). In the food sector, however, DMY applications are hindered by inherent physicochemical limitations, including sensitivity to light, heat, and pH, which lead to rapid oxidative degradation. Furthermore, its direct bioavailability is below 4% [25], and as reported by Fan et al. [26], its aqueous solubility at 25 °C and 37 °C is merely 0.2 mg/mL and 0.9 mg/mL, respectively. Electrospinning offers a robust strategy to overcome these bottlenecks. By providing a high specific surface area and porosity, this technique ensures the molecular-level dispersion and efficient encapsulation of DMY. The resulting carrier–active interactions significantly enhance DMY’s stability and enable controlled release behavior [27,28,29]. Evidence shows that electrospun encapsulation can achieve efficiencies exceeding 90%, while substantially preserving the thermal stability and antioxidant activity of DMY, suggesting its potential to provide useful information for the future design of active packaging materials [30].

The present study constructed a ternary composite nanofiber system of PVA/gelatin/zein as a delivery carrier for DMY, aiming to further enhance the biopolymer compatibility and mechanical properties of the encapsulation system, thereby exploring the feasibility of using PVA/gelatin/zein nanofibers as a carrier system for DMY and evaluating their physicochemical properties, release behavior, antioxidant activity, and antibacterial activity against representative bacteria. Scanning electron microscopy (SEM) was used to characterize the morphology and diameter distribution of the nanofibers. The possible intermolecular interactions among DMY, gelatin, zein, and PVA were investigated using a combination of techniques including Fourier transform infrared (FTIR) spectroscopy, thermal analysis, and water contact angle (WCA) measurements. Furthermore, the composite fibrous membranes were subjected to in vitro antioxidant and antibacterial performance tests. This study is expected to provide preliminary material-design information for DMY-loaded PVA/gelatin/zein nanofibrous membranes, while further application-oriented validation remains necessary.

## 2. Materials and Methods

### 2.1. Chemicals

Poly(vinyl alcohol) (PVA), gelatin, acetic acid, and dihydromyricetin (DMY) were purchased from Aladdin Bio-Chem Technology Co., Ltd. (Shanghai, China). Zein (Product No. Z3625), 2,2-diphenyl-1-picrylhydrazyl (DPPH), 2,2′-azino-bis (3-ethylbenzothiazoline-6-sulfonic acid) diammonium salt (ABTS), and 2,4,6-tri(2-pyridyl)-1,3,5-triazine (TPTZ) were obtained from Sigma-Aldrich (St. Louis, MO, USA). All other chemical reagents used in this study were of analytical grade and produced in China.

### 2.2. Solution of Electroblowing Spinning

**Figure 1:**
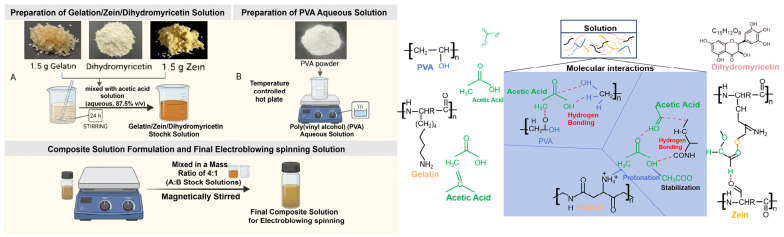
Flow chart of the preparation of DMY/PVA/gelatin/zein blended spinning solutions.

The electroblowing spinning solutions were prepared as follows (Figure 1): 1.5 g of gelatin and 1.5 g of zein were dissolved in 10 mL of 87.5% (*v*/*v*) acetic acid solution under magnetic stirring until a homogeneous mixture was obtained. Subsequently, DMY was incorporated into the solution at concentrations of 20%, 22.5%, 25%, and 27.5% (*w*/*w*) relative to the total protein mass; these dosages were determined based on preliminary experiments. Separately, 1.0 g of PVA was dissolved in 10 mL of deionized water and heated in a 90 °C water bath until complete dissolution, yielding a 10% (*w*/*v*) PVA aqueous solution. Finally, the gelatin/zein/DMY solution and the PVA solution were blended at a mass ratio of 4:1 and stirred continuously for 24 h to ensure thorough mixing and uniform dispersion of all components.

### 2.3. Fiber Spinning

**Figure 2:**
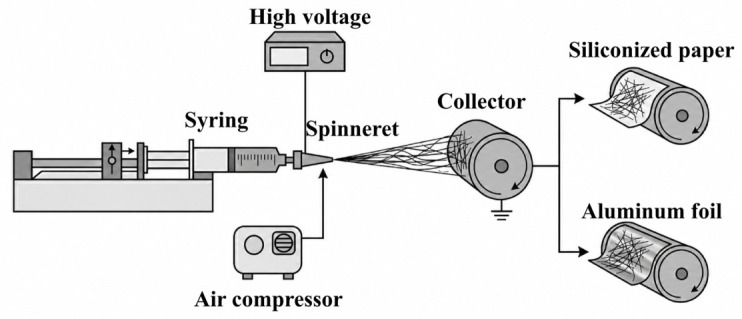
Schematic of electroblowing spinning equipment.

The configuration of the electroblowing spinning system employed in this study is illustrated in Figure 2, adapted from previous literature in our group [30]. Nanofibrous membranes were fabricated using a JDF05 electroblowing spinning apparatus (NaYi Instrument, Changsha, China). All electroblowing spinning experiments were conducted under controlled environmental conditions at a temperature of 25 ± 2 °C and a relative humidity of 50 ± 5%. The core components of the apparatus integrated a high-voltage generator, a precision syringe pump, an aerodynamic compressor, and a grounded stainless steel rotating drum collection system. During experimental operations, the prepared homogeneous precursor solution was transferred into a 10 mL polypropylene syringe equipped with a corresponding stainless-steel nozzle. The electroblowing spinning process was subsequently initiated by applying an electrostatic voltage of 20 kV and a constant infusion rate of 10 mL/h, with the tip-to-collector distance fixed at 15 cm. Furthermore, to optimize jet dynamics and enhance fiber formation quality, an auxiliary airflow was maintained at a constant rate of 350 L/h via the air pump. Nanofibers intended for routine characterization were collected on siliconized paper at ambient temperature, while those designated for antibacterial performance testing were collected on aluminum foil under identical conditions. The resulting nanofibers contained DMY at mass ratios of 0%, 20%, 22.5%, 25%, and 27.5% relative to the total protein mass; these samples were designated as D1, D2, D3, D4, and D5, respectively.

### 2.4. Rheological Measurement

Rheological characterization of the spinning solution was performed at 25 °C with a rotational rheometer (TA Instruments AR2000ex, TA Instruments, New Castle, DE, USA). A 1.5 mL aliquot of the sample was loaded into a 40 mm parallel-plate geometry. Three types of measurements were carried out: (1) steady-state shear tests (shear rate: 1–200 s^−1^) to examine the correlation between shear stress and apparent viscosity; (2) strain sweep tests (strain: 0.001–100%, angular frequency: 6.283 rad/s) to identify the linear viscoelastic regime and assess the storage modulus (G′) and loss modulus (G″); and (3) dynamic frequency sweep tests (frequency: 0.1–100 rad/s, strain: 1%) to further probe the viscoelastic behavior of the solution.

### 2.5. Fiber Morphologies

Before the experiment, a small amount of the sample was fixed on conductive adhesive and subjected to vacuum treatment. The surface morphology was then observed using a scanning electron microscope (Gemini SEM 300, ZEISS, Oberkochen, Baden-Württemberg, Germany) at an accelerating voltage of 3 kV and a magnification of 5000×. For each sample, 100 fibers were randomly chosen, and their diameters were measured and statistically analyzed with Nano Measurer 1.2 software.

### 2.6. Fourier Transform Infrared (FTIR) Analysis

FTIR analysis was carried out using a Thermo Fisher Scientific Nicolet iS20 spectrometer (Thermo Fisher Scientific, Madison, WI, USA) over the range of 4000–400 cm^−1^ at a spectral resolution of 4 cm^−1^, obtaining 32 scans per measurement. Before each measurement, a background spectrum was collected using air for background correction. The sample was mixed with potassium bromide (KBr) and pressed into a pellet; the acquired spectral data were processed and analyzed with OMNIC 8.0 and Origin 2026 software.

### 2.7. X-Ray Diffraction (XRD) Analysis

X-ray diffraction (XRD) analysis was conducted on a Rigaku SmartLab SE diffractometer (Tokyo, Japan) equipped with a Cu Kα radiation source (λ = 1.5406 Å). The diffractometer was operated at a voltage of 40 kV and a current of 200 mA, with data collected over a 2θ range of 10° to 80° at a scanning rate of 1°/min. All measured data were subsequently processed and analyzed using Origin 2026 software (OriginLab, Northampton, MA, USA).

### 2.8. Thermal Analysis

The thermal analysis of the fibers was investigated by differential scanning calorimetry (DSC) and thermogravimetric analysis (TGA). Approximately 5 mg of the fiber sample was heated under nitrogen atmosphere from 30 °C to 615 °C at a ramp rate of 10 °C/min. The resulting data were post-processed and analyzed with Origin 2026 software.

### 2.9. Water Contact Angle (WCA) Measurement

The WCA of the fibers was assessed using a Chengde Dingsheng JY-82C video-based contact angle meter (Chengde, China). The sample was placed flat onto a glass slide, and a droplet of deionized water was applied to the fiber surface. The dynamic evolution of the droplet was recorded in real time at ambient temperature. All measurements were performed in triplicate for each sample to ensure the reliability of the data.

### 2.10. Water Vapor Permeability (WVP) Analysis

10 mL of deionized water was added to a water vapor permeability test cup, which was then sealed with a fibrous membrane of 6 cm in diameter and uniform thickness (the thickness was measured using a digital thickness gauge). After recording the initial mass, the cup was placed in a desiccator maintained at 28 °C and 0% relative humidity. Over the subsequent 6 h, the mass of the cup was weighed at 1-h intervals to monitor water vapor permeation:

$$WVP\left(g/m\cdot s\cdot Pa\right)=\frac{Ws\times L}{t\times A\times \Delta P}$$

In this equation, A represents the effective exposed area of the nanofibrous membrane (cm^2^), and L denotes the membrane thickness (cm). ΔP refers to the water vapor pressure difference (Pa), calculated based on the saturated water vapor pressure at 28 °C (2237.8 Pa). Ws/t corresponds to the slope of the linear regression of mass change versus time (g·s^−1^), representing the steady-state water vapor transmission rate.

### 2.11. Mechanical Properties

The tensile strength (TS), elongation at break (EB), and elastic modulus (EM) of the nanofibers were determined using a DR-508A automated tensile testing machine (Dongri Instrument Ltd., Dongguan, China). The samples were cut into rectangular strips with dimensions of approximately 10 mm × 3 mm for testing. The thickness of each specimen was measured at multiple locations using a digital micrometer, and the average value was recorded. The initial gauge length was set to 20 mm, and tensile tests were conducted at a crosshead speed of 5 mm·min^−1^. Prior to testing, all samples were conditioned at 25 ± 2 °C and 50 ± 5% relative humidity for 24 h to ensure equilibrium and stable moisture content. Five replicate measurements were performed for each sample to ensure the accuracy and reproducibility of the results.

### 2.12. Release of Dihydromyricetin (DMY)

The in vitro release behavior of DMY from the composite nanofibrous membrane was investigated using a shaking method under sink conditions. Due to the limited aqueous solubility of DMY, a 20% (*v*/*v*) aqueous methanol solution was selected as the release medium to enhance DMY solubility and prevent premature saturation during the release test. This medium was employed as a model solvent to provide controlled and reproducible release conditions rather than to directly simulate actual food systems or standardized food simulants. Accurately weighed samples of DMY-loaded nanofibrous membrane were placed into conical flasks containing 5 mL of release medium (20% (*v*/*v*) methanol aqueous solution). The flasks were then incubated in a thermostatic shaker at 37 °C and 150 rpm, protected from light. At predetermined time intervals (20 min, 40 min, 1 h, 2 h, 4 h, 6 h, 12 h, 24 h, 48 h, and 72 h), 1 mL of release medium was precisely withdrawn and immediately replenished with an equal volume (1 mL) of fresh release medium pre-warmed to 37 °C to maintain a constant medium volume. The collected samples were filtered through a 0.22 μm membrane filter and analyzed by HPLC. To verify sink conditions, the maximum theoretical concentration of DMY in the release medium was calculated based on the highest DMY loading (27.5 wt%) and sample mass (2.865 mg), resulting in a maximum concentration of 0.1576 mg/mL. Considering that the solubility of DMY in hydroalcoholic media has been reported to be substantially higher than that in pure water, this concentration is expected to remain well below the saturation limit under the present experimental conditions.

The concentration of DMY in the release medium was determined by high-performance liquid chromatography (HPLC). The chromatographic conditions were as follows: column, C_18_ reversed-phase column (4.6 × 150 mm, 3 μm); mobile phase, 0.1% phosphoric acid aqueous solution (A)–methanol (B); gradient elution program as shown in Table 1; flow rate, 0.5 mL/min; column temperature, 25 °C; detection wavelength, 292 nm; injection volume, 10 μL.

A DMY reference standard was accurately weighed and dissolved in the release medium (20% (*v*/*v*) methanol aqueous solution) to prepare a stock solution, which was then serially diluted to obtain a series of standard solutions of different concentrations. The mass concentration of DMY (x, μg/mL) was used as the abscissa and the corresponding peak area (y) as the ordinate. Linear regression analysis yielded the regression equation y = 35980x − 646,443 (R^2^ = 0.9992).

To further investigate the release mechanism of DMY from the nanofibrous membranes, the cumulative release data were fitted to zero-order, first-order, and Higuchi kinetic models. The mathematical expressions of these models are shown below:

Zero-order model:

M_t_ = k_0_t

First-order model:

ln(1 − M_t_/M_∞_) = −k_1_t

Higuchi model:

M_t_/M_∞_ = k_H_t^1/2^

where M_t_ is the cumulative amount of DMY released at time t, M_∞_ is the cumulative amount released at equilibrium, and k_0_, k_1_, and k_H_ are the release rate constants of the zero-order, first-order, and Higuchi models, respectively.

Linear regression analysis was performed using Origin 2026 software (OriginLab, USA), and the goodness of fit was evaluated by the coefficient of determination (R^2^). The model exhibiting the highest R^2^ value was considered to provide the most appropriate description of the release behavior.

### 2.13. Antioxidant and Antibacterial Activity

The antioxidant capacity of the nanofiber membranes was evaluated by free radical scavenging (DPPH and ABTS) and metal ion reducing (Fe^3+^/Cu^2+^) assays, following the procedures reported by Liu et al. [31] with minor modifications. All experiments were performed in triplicate. In the DPPH assay, 1 mg of fiber membrane containing different amounts of DMY was mixed with 2 mL of DPPH solution and incubated in the dark for 30 min, after which the absorbance was measured at 517 nm. For the ABTS assay, a mixture of ABTS solution and K_2_S_2_O_8_ solution was first allowed to react in the dark for 12 h, then diluted with phosphate buffer to an absorbance of 0.7 ± 0.02 at 734 nm. Subsequently, 1 mg of the sample was added to 2 mL of this working solution, incubated in the dark for another 30 min, and the absorbance was recorded at 734 nm. In the Fe^3+^ reducing assay, a TPTZ working solution was freshly prepared by mixing 20 mmol/L FeCl_3_ solution, TPTZ stock solution, and 0.3 mol/L sodium acetate buffer at a volume ratio of 1:1:10. A 1 mg sample was combined with 2 mL of the working solution, kept at 37 °C for 30 min, and the absorbance was read at 592 nm. For the Cu^2+^ reducing assay, equal volumes of 0.01 mol/L Cu^2+^ solution, 1 mol/L CH_3_COONH_4_ solution, and 7.5 mmol/L neocuproine (C_14_H_12_N_2_) solution were mixed. The fiber membrane (1 mg) was introduced into 2 mL of this mixture, incubated at 25 °C for 30 min, and the absorbance was determined at 450 nm. DPPH and ABTS results were expressed as radical scavenging rates, whereas Fe^3+^ and Cu^2+^ reducing capacities were expressed as absorbance values. All antioxidant activity assays were performed using the nanofibrous membranes in their intact solid state. Membrane specimens were cut into appropriately sized pieces and directly immersed into the test solutions without prior dissolution or dispersion.

The antibacterial activity of the nanofibers was assessed by the inhibition zone method against *Escherichia coli* (*E. coli*) and *Staphylococcus aureus* (*S. aureus*), based on the method described by Liu et al. [32] with slight adjustments according to the experimental conditions. The diameter of the inhibition zone was measured using a vernier caliper, and all tests were carried out in triplicate to ensure the reliability of the results. For the antibacterial assays, the nanofibrous membranes were tested as intact solid films. Membrane disks of a defined diameter were placed directly onto the inoculated agar plates without any prior dissolution or dispersion treatment.

### 2.14. Statistical Analysis

One-way analysis of variance (ANOVA) was used to assess statistical significance, with Tukey’s post hoc test applied to identify specific differences among samples. A *p*-value of less than 0.05 was regarded as statistically significant. All statistical analyses and curve plotting were carried out using Origin 2026 software (OriginLab, USA).

## 3. Results and Discussion

### 3.1. Rheological Measurements

**Figure 3:**
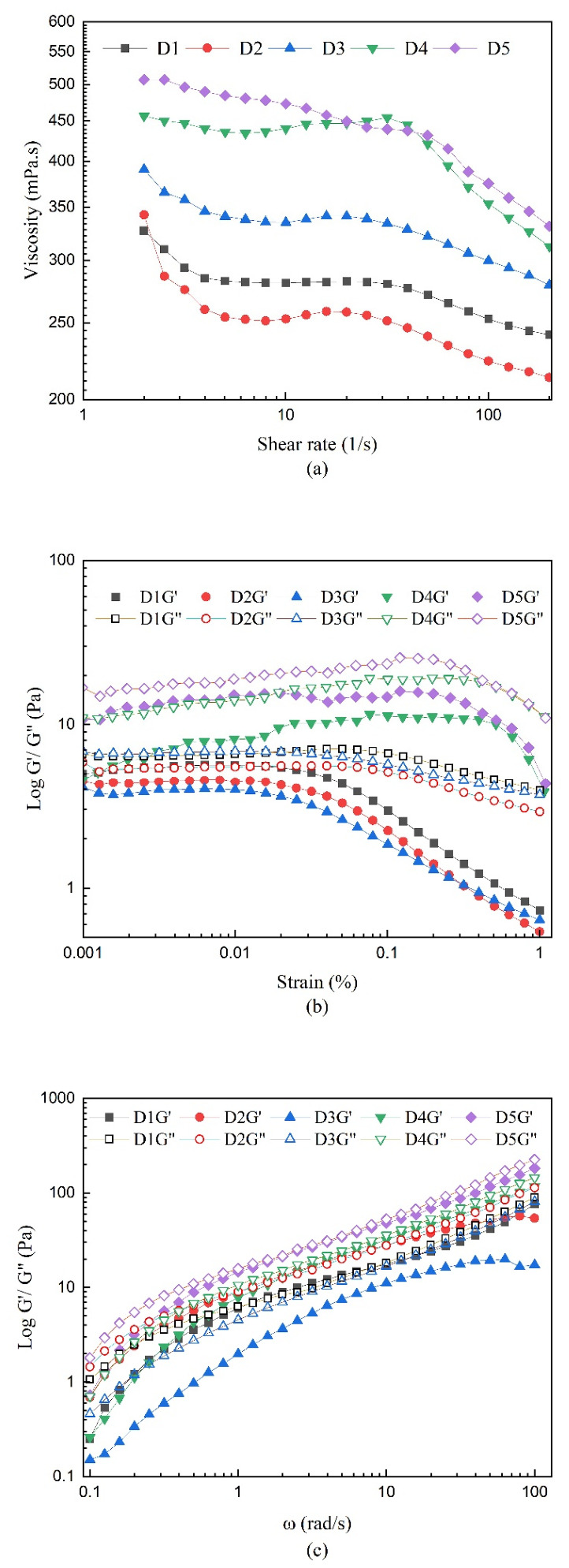
Rheological behavior of PVA/gelatin/zein composite solutions loaded with 0%, 20%, 22.5%, 25%, and 27.5% DMY: (**a**) viscosity; (**b**) linear viscoelastic region; (**c**) dynamic frequency sweep.These samples were designated as D1, D2, D3, D4, and D5, respectively.

Figure 3a–c presents the influence of varying DMY content on the rheological behavior of the PVA/gelatin/zein composite system, including steady-state flow behavior (apparent viscosity), strain sweep (linear viscoelastic region), and dynamic frequency sweep characteristics. Table 2 summarizes the flow behavior index (n) and consistency coefficient (K) obtained by fitting the power-law model, aiming to elucidate the regulatory role of DMY in the structure–property relationships governing the flow state and viscoelasticity of the system from the perspective of molecular interactions. The apparent viscosity of all formulations decreased monotonically with increasing shear rate, indicating pronounced shear-thinning behavior, i.e., typical pseudoplastic fluid characteristics. As shown in Table 2, the neat PVA/gelatin/zein system (D1) displayed a relatively high viscosity (K = 0.41 ± 0.06), which is primarily attributed to the strong hydrogen-bonding network formed between the abundant hydroxyl groups on PVA segments and protein molecules, as well as the solvation effect of water molecules with polymer chains that restricts the segmental motion of macromolecules [33,34].

Notably, as the DMY mass fraction increased, the system viscosity (characterized by the K) exhibited a non-monotonic trend, i.e., an initial decrease followed by a subsequent increase. Specifically, in D2 and D3, the K values progressively declined relative to the base system D1, suggesting that the introduction of DMY at low concentrations may have disrupted the originally stable hydrogen-bonding network between PVA and protein molecules, exerting a “plasticizing” or “diluting” effect and thereby weakening the overall structural integrity of the system. Notably, despite the decrease in K values at low DMY loadings (D2 and D3), the apparent viscosity across the measured shear rate range remained consistently higher than that of the neat system (D1), as shown in Figure 3a. The reduced K values at these loadings primarily reflect the more pronounced shear-thinning behavior (i.e., lower n values) rather than an actual decrease in viscosity. However, when the DMY concentration was further increased to D4 and D5, the K values rebounded significantly and ultimately surpassed that of the base system. This indicates that the role of DMY as a “molecular bridging agent” becomes dominant at high concentrations: as a natural flavonoid rich in phenolic hydroxyl groups, DMY can generate extensive physical crosslinking with PVA and protein backbones through multiple hydrogen bonds and hydrophobic interactions, constructing a more compact spatial micro-network and consequently enhancing the structural strength of the system considerably [35,36]. Furthermore, the flow behavior index n of all samples was less than 1 (0.88–0.92), indicating that the incorporation of DMY did not alter the non-Newtonian nature of the fluids. Nevertheless, the n value exhibited a gradual downward trend with increasing DMY concentration. This subtle decrease reflects the heightened sensitivity to shear stress of the physical crosslinked network formed at high DMY loadings under steady shear, further corroborating the network-strengthening effect of DMY. In electroblowing spinning, the relatively higher apparent viscosity observed at elevated DMY loadings (D4 and D5) may enhance the resistance of the jet to elongational deformation, suppressing jet breakup and promoting the formation of more uniform fibers.

The strain sweep curves (Figure 3b) show that in the low-strain region, both the storage modulus (G′) and loss modulus (G″) remained at a high-level plateau, indicating that the system lies within the linear viscoelastic region (LVE) and that the internal microstructure possesses good mechanical stability. When the strain exceeded a critical value, G′ and G″ decreased abruptly, signifying that the externally imposed deformation disrupted the physical crosslinking network established between DMY and biomacromolecules, leading to structural breakdown of the system.

Figure 3c illustrates the effect of dynamic angular frequency (ω) on the moduli. Over the tested frequency range, both G′ and G″ increased with rising frequency, displaying a pronounced frequency dependence. Noteworthily, across the entire frequency sweep range, the loss modulus G″ was consistently higher than the storage modulus G′ (i.e., loss tangent tan δ > 1), indicating that although molecular chain entanglements and DMY-induced interactions exist in the system, the viscoelastic response is predominantly viscous over the examined time scale, exhibiting a sol-like or “liquid-like” rheological behavior [37]. This viscous dissipation-dominated rheological characteristic endows the composite solution with excellent extensional deformation capability during electrospinning, allowing the molecular chains to undergo orientational rearrangement along with the electric field force within an extremely short time.

### 3.2. Nanofiber Morphology

**Figure 4:**
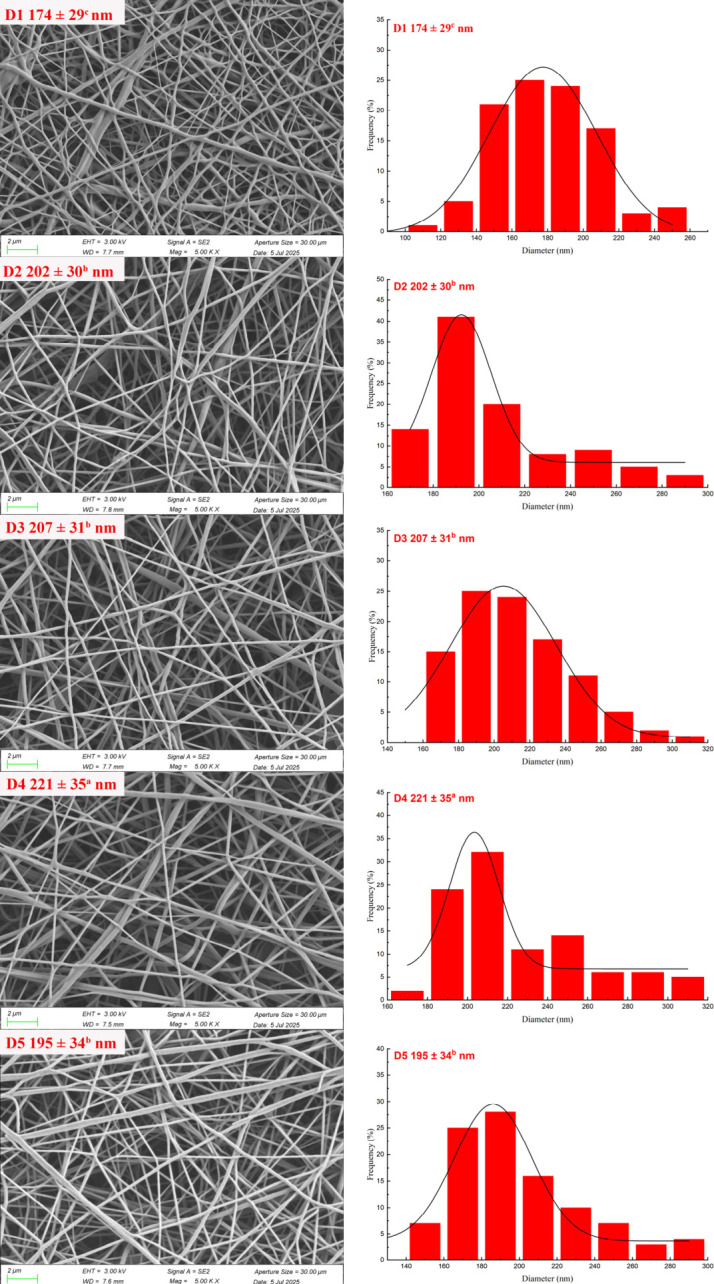
SEM micrographs and fiber diameter distributions of PVA/gelatin/zein nanofibers loaded with 0%, 20%, 22.5%, 25%, and 27.5% DMY; these samples were designated as D1, D2, D3, D4, and D5, respectively.

As shown in Figure 4, the SEM images and fiber diameter distributions of PVA/gelatin/zein composite nanofiber membranes prepared with different DMY loading concentrations exhibit a distinct composition-dependent morphological evolution. Upon the incorporation of DMY, the fibers become more uniform, straighter, and more regularly arranged. This phenomenon may be attributed to the formation of intermolecular hydrogen bonds between the multiple phenolic hydroxyl groups of DMY and the polar groups on the polymer chains (especially those of gelatin and zein), which increases the cohesive energy density of the solution, thereby suppressing the whipping instability of the jet during electrospinning and facilitating the formation of more regular fibers [38].

With the introduction of DMY, the average fiber diameter increased from 174 nm to 221 nm. This trend is generally consistent with the rheological results, which showed that the apparent viscosity of the DMY-containing solutions remained higher than that of the neat PVA/gelatin/zein system across the investigated shear-rate range, despite the non-monotonic variation in the K. The increase in apparent viscosity can enhance the resistance of the jet to elongational deformation, thereby reducing jet thinning and resulting in larger fiber diameters. A similar positive correlation between solution viscosity and fiber diameter has been reported by Liu et al. [39] in the PLGA-HA system. However, it should be noted that the relationship between solution properties and fiber diameter is not universally governed by a single parameter. The evolution of nanofiber diameter is a complex process influenced by the synergistic interplay of multiple factors, including viscosity, polymer molecular weight, electrical conductivity, and surface tension. The addition of high-molecular-mass components such as dextran has been shown to decrease rheological properties while simultaneously increasing fiber diameter, contrary to the viscosity-dominated trend [40]. In our specific PVA/gelatin/zein/DMY system, the observed increase in fiber diameter is predominantly driven by the enhanced solution viscosity resulting from DMY-mediated intermolecular hydrogen bonding, as supported by the rheological data. These observations underscore that the control of fiber morphology requires a comprehensive consideration of the specific physicochemical properties of the incorporated additives and the resulting changes in multiple solution parameters.

Notably, when the DMY loading reached the highest level (27.5%), the average fiber diameter decreased to 195 nm despite the highest K and apparent viscosity observed in the rheological measurements. This non-monotonic behavior suggests that factors other than viscosity may also influence fiber formation at high DMY loadings. A possible explanation is that excessive DMY may undergo aggregation or partial crystallization within the polymer matrix, which could affect the homogeneity of the spinning solution and consequently alter jet stretching behavior during electrospinning. As a result, a reduction in the average fiber diameter was observed at D5. Nevertheless, further investigations would be required to clarify the underlying mechanism.

### 3.3. FTIR Spectra Analysis

**Figure 5:**
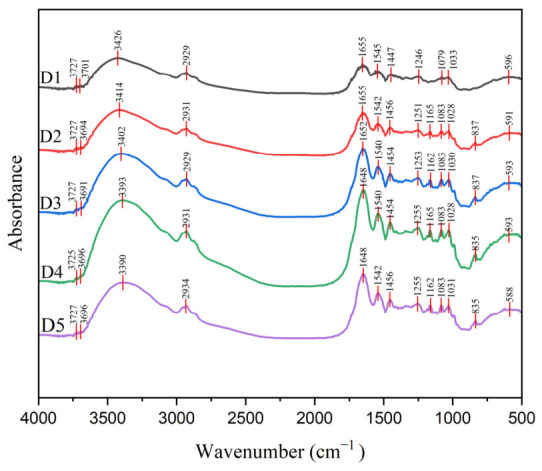
FTIR spectra of PVA/gelatin/zein nanofibers loaded with 0%, 20%, 22.5%, 25%, and 27.5% DMY; these samples were designated as D1, D2, D3, D4, and D5, respectively.

The functional group characteristics and possible intermolecular interactions of the PVA/gelatin/zein nanofibrous membranes with different DMY loadings were investigated by FTIR spectroscopy (Figure 5). In the 3600–3000 cm^−1^ region, the broad absorption band centered at approximately 3426 cm^−1^ is assigned to the stretching vibrations of O–H (from PVA, water, and phenolic hydroxyls) and N–H groups (from gelatin and zein). Upon the incorporation of DMY, this band exhibited a distinct redshift from 3426 cm^−1^ (D1) to 3390 cm^−1^ (D5), accompanied by noticeable peak broadening. This spectral evolution suggests the formation of intermolecular hydrogen bonds between the phenolic hydroxyl groups of DMY and the polar moieties of the polymeric matrix [41]. Furthermore, the Amide I band of the proteins, corresponding to C=O stretching, shifted from 1655 cm^−1^ in D1 to 1648 cm^−1^ in the D4/D5, which is consistent with enhanced hydrogen bonding between DMY and the peptide linkages, although a subtle contribution from protein secondary structural perturbations cannot be excluded.

In the fingerprint region, significant peak shifts and the emergence of new characteristic bands were observed. With increasing DMY concentration, the absorption peak attributed to –CH_2_– bending vibrations gradually shifted from 1447 cm^−1^ to 1456 cm^−1^, suggesting changes in the local molecular environment arising from enhanced intermolecular interactions [42]. Concurrently, the Amide III band (attributed to C–N stretching and N–H bending) moved from 1246 cm^−1^ to 1255 cm^−1^, indicating a rearrangement of the hydrogen-bonding network induced by DMY [38], which aligns well with the aforementioned redshift of the O–H stretching band.

The incorporation was further supported by the emergence of distinctive peaks at 1165 cm^−1^ and 837 cm^−1^, absent in the pristine nanofibers (D1). The peak at 1165 cm^−1^ is attributed to the C–O stretching vibration associated with the phenolic hydroxyl groups of DMY [43], while the sharp peak at 837 cm^−1^ corresponds to the out-of-plane C–H bending vibrations of the DMY aromatic rings [44].

The FTIR results suggest that DMY was incorporated into the polymeric matrix and may interact with the matrix through hydrogen bonding. These interactions are likely to contribute to the modification of the local molecular environment and may partially account for the enhancement in mechanical properties observed in the composite nanofibrous membranes.

### 3.4. XRD Analysis

**Figure 6:**
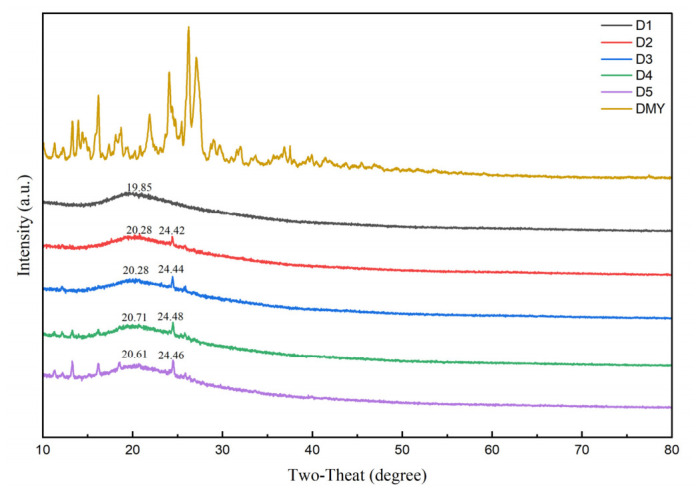
XRD spectra of PVA/gelatin/zein nanofibers loaded with 0%, 20%, 22.5%, 25%, and 27.5% DMY, these samples were designated as D1, D2, D3, D4, and D5, respectively.

The effect of DMY on the crystal structure of PVA/gelatin/zein nanofibers was investigated by XRD (Figure 6). According to Geng et al. [45], pure DMY exhibits distinct crystalline diffraction peaks in the range of 10–30°. Comparison with the XRD pattern of pure DMY reveals that it has no characteristic diffraction peak near 20.6°, and therefore the gradual shift in the main diffraction peak center from 19.85° to 20.61° for the DMY-loaded fibers is not attributable to the superposition of DMY crystalline peaks. This shift corresponds to a reduction in the average interchain spacing of the polymer matrix, suggesting a change in molecular packing after DMY incorporation. Such behavior may be associated with the intermolecular interactions between DMY and the polymer components, which is consistent with the shift trends of the O–H/N–H characteristic peaks observed in FTIR [46].

Notably, a new diffraction peak appears near 24.42° for the DMY-loaded fibers, and its intensity increases continuously with increasing DMY loading. This observation suggests the presence of DMY-associated ordered domains within the nanofibrous matrix. However, based solely on diffraction peak intensity, it is not possible to conclusively determine crystal growth, crystallinity enhancement, or the evolution of ordered aggregates. Therefore, the observed increase in peak intensity is interpreted as evidence of an increased contribution of ordered DMY-related structures within the fibers. For D5, the diffraction peak at 24.42° becomes more pronounced. Combined with the SEM observations showing reduced fiber uniformity and a broader diameter distribution, this result may suggest that excessive DMY loading affects the structural homogeneity of the composite system, thereby influencing fiber formation during electroblowing spinning.

### 3.5. Thermal Characteristics

**Figure 7:**
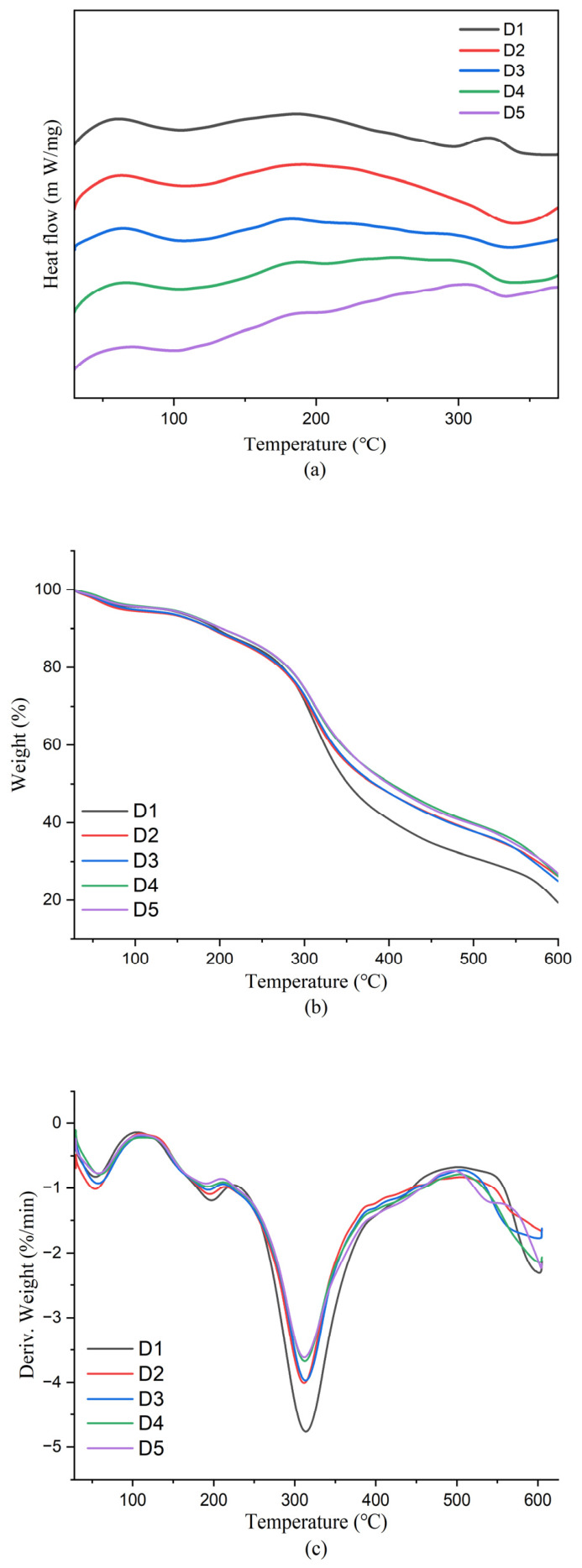
DSC (**a**), TGA (**b**), and DTG (**c**) curves of PVA/gelatin/zein nanofibers loaded with 0%, 20%, 22.5%, 25%, and 27.5% DMY; these samples were designated as D1, D2, D3, D4, and D5, respectively.

Figure 7 presents DSC (a), TGA (b), and derivative thermogravimetry (DTG, c) curves of PVA/gelatin/zein nanofibers with different DMY loadings, and the corresponding data are summarized in Table 3. All samples exhibited similar thermal transition profiles, indicating that the incorporation of DMY did not fundamentally alter the thermal behavior of the PVA/gelatin/zein matrix. No distinct melting peak corresponding to crystalline DMY was observed, suggesting that crystalline DMY, if present, was either highly dispersed, partially amorphized, or below the detection limit of DSC. With increasing DMY content, slight variations in the DSC curves were observed, particularly for D5. These changes may be associated with enhanced intermolecular interactions, such as hydrogen bonding between DMY and the polymer chains, which can influence chain mobility and thermal relaxation behavior. Overall, the DSC results indicate that DMY incorporation affects molecular interactions within the nanofibrous membranes without producing clear evidence of substantial crystallinity enhancement.

The TGA curves of the fibers exhibit three distinct degradation stages. The first stage of mass loss occurs at approximately below 100 °C, which is primarily attributed to the evaporation of adsorbed moisture from the samples. The second degradation stage appears at around 200 °C, corresponding to the thermal decomposition of the protein components. The third stage takes place at approximately 312 °C with the maximum mass loss rate, which is a complex degradation region where multiple decomposition processes may overlap, including the further degradation of PVA, protein backbone breakdown (gelatin and zein), and the formation of carbonaceous residues from the composite system. Therefore, this stage cannot be attributed to a single component and is more appropriately considered as a combined decomposition and carbonization process of the multicomponent nanofibers. Similar thermal decomposition behavior was observed by Deng et al. [47] in glucose-crosslinked gelatin/zein nanofibers encapsulating allopurinol.

The abundant hydroxyl groups (–OH) on the PVA molecular chains can form numerous intermolecular hydrogen bonds with the carboxyl (–COOH), amino (–NH_2_), or hydroxyl groups in gelatin and zein, thereby enhancing the structural stability of the system [33]. With increasing DMY content, the thermal decomposition temperatures of the PVA/gelatin/zein nanofibers in the second and third stages gradually decreased, indicating a decline in the overall thermal stability of the system, which was particularly pronounced at high DMY concentrations. FTIR and XRD analyses suggested the possible formation of hydrogen-bonding interactions and changes in molecular organization between DMY and the polymer matrix, promoting closer chain packing. However, as DMY loading continues to increase, excessive small-molecule DMY may partially disrupt the original hydrogen bonding network and reduce the effective chain entanglement density among polymer chains, thereby influencing the thermal stability of the composite system. This effect becomes particularly significant at a high DMY concentration (27.5%, D5), where SEM observations revealed deteriorated fiber morphology and broadened diameter distribution, and XRD detected an enhanced contribution of DMY-associated ordered domains. Taken together, the influence of excessive DMY on the structural homogeneity of the polymer network may be the primary cause of the slight decline in thermal stability.

### 3.6. WCA

**Figure 8:**
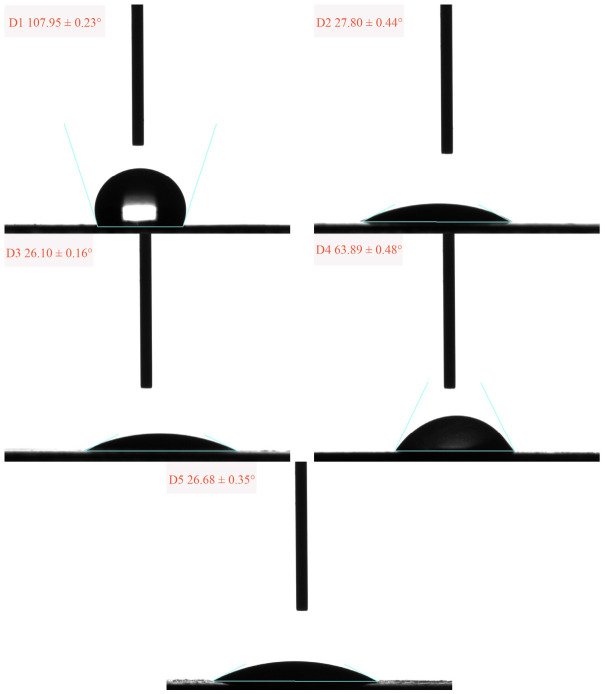
WCA of PVA/gelatin/zein nanofibers loaded with 0%, 20%, 22.5%, 25%, and 27.5% DMY; these samples were designated as D1, D2, D3, D4, and D5, respectively.

The hydrophilicity of electrospun nanofibrous membranes plays a crucial role in regulating the release behavior of bioactive substances. To evaluate the surface wettability of the composite nanofibrous membranes, water contact angle (WCA) measurements were performed. As shown in Figure 8, the WCA decreased significantly from 107.95° ± 0.23° to 26.68° ± 0.35° with increasing DMY content. Generally, a WCA greater than 90° indicates a hydrophobic surface, whereas a value below 90° reflects good hydrophilicity [48]. These results demonstrate that the incorporation of DMY markedly enhanced the surface hydrophilicity of the membranes, which may be primarily attributed to the abundant phenolic hydroxyl groups present in DMY molecules: these hydroxyl groups can form hydrogen bonds with water molecules, thereby improving the water absorption capacity of the membranes. This observation is consistent with previous reports; for instance, the introduction of DMY into PVP/chitosan nanofiber systems also significantly improved the hydrophilicity of the materials [32].

Notably, when the DMY concentration reached 25% (D4), the WCA increased to 63.89° ± 0.48°, indicating a temporary reduction in hydrophilicity. However, upon further increasing the DMY concentration to 27.5% (D5), the WCA decreased sharply to 26.68° ± 0.35°. This non-linear variation may be related to changes in the distribution and aggregation state of DMY within the nanofibrous matrix. The XRD results indicated the formation of more ordered DMY structures at higher loading levels. Therefore, it is possible that interactions between DMY and the polymer matrix, together with changes in DMY organization at different concentrations, influenced the exposure of hydrophilic groups on the fiber surface and consequently affected the wettability of the membranes [49]. When the DMY content was further increased to 27.5% (D5), additional DMY molecules may have become more accessible at or near the fiber surface, resulting in enhanced surface hydrophilicity and a lower WCA. However, further studies using more direct characterization techniques would be required to fully elucidate the underlying mechanism.

From a food-packaging perspective, the wettability of the nanofibrous membranes may influence moisture management, surface hydration, and the release behavior of incorporated bioactive compounds. The enhanced hydrophilicity observed in most DMY-loaded membranes may facilitate interactions with aqueous environments and influence the hydration behavior of the membrane, which could be relevant to the release of incorporated bioactive compounds and the overall performance of food-contact materials. However, excessively high hydrophilicity may also increase water absorption, which could potentially affect the dimensional stability and mechanical integrity of the packaging material under humid conditions. Therefore, the non-linear variation in WCA observed at different DMY loadings suggests that the balance between wettability and structural stability should be carefully considered when designing food-contact materials. Nevertheless, further application-oriented studies, such as moisture permeability and food-storage evaluations, are required to determine the optimal DMY loading for practical food-packaging applications.

### 3.7. WVP

**Figure 9:**
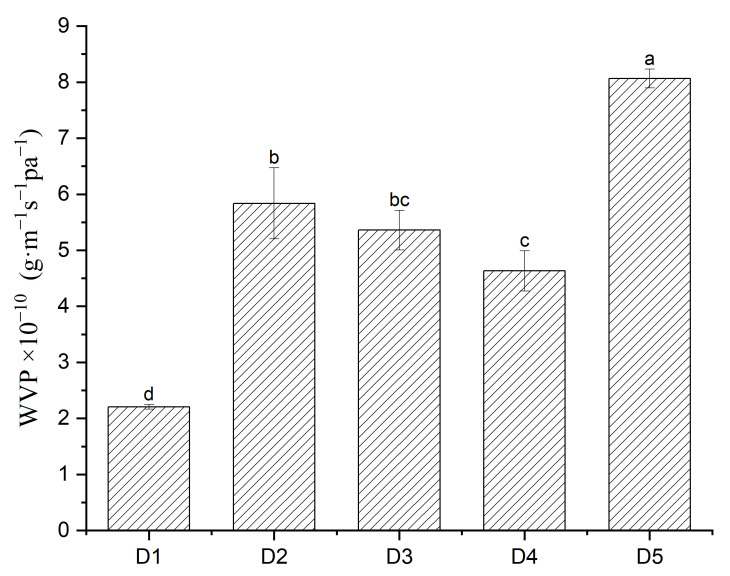
Water vapor permeability of PVA/gelatin/zein nanofibers loaded with 0%, 20%, 22.5%, 25%, and 27.5% DMY; these samples were designated as D1, D2, D3, D4, and D5, respectively. Different superscripts within the same column indicate values that differ significantly (*p* < 0.05).

Water vapor permeability (WVP) is a key parameter that determines the moisture barrier performance of nanofibrous membranes and the overall effectiveness of active packaging systems. The test results are shown in Figure 9. The pristine PVA/gelatin/zein membrane exhibited a relatively low WVP, whereas the introduction of DMY led to a significant increase in WVP. One possible explanation is that at low DMY loading, the phenolic hydroxyl groups in DMY molecules may interact with the polymer chains through hydrogen bonding, thereby altering the intermolecular interaction network and facilitating water vapor transport through the membrane. At moderate DMY concentrations, the formation of DMY-associated ordered structures may partially restrict water vapor diffusion by increasing the tortuosity of transport pathways. As indicated by XRD analysis, the contribution of DMY-associated ordered domains increases with DMY loading. At moderate DMY concentrations, these ordered domains, together with the hydrogen bonding network, may create a more tortuous pathway for water vapor diffusion, which could partially restrict transport and contribute to the observed slight decrease in WVP. However, when the DMY content reached the D5 level, the WVP increased significantly again. Based on the SEM observations and XRD results, this increase may be associated with structural heterogeneity arising from excessive DMY loading. Such structural changes could potentially create additional pathways for water vapor transport, thereby facilitating water vapor permeation. This transition is corroborated by the deterioration of fiber morphology observed by SEM in D5 and the sharp decrease in WCA. Similarly, Cabrera-Barja et al. [50] reported that when fungal chitosan nanofibers (ChNF) were incorporated into hydroxypropyl methylcellulose-based active packaging films, a high ChNF content resulted in a “layered/discontinuous phase” structure containing more microvoids or defects, which in turn increased the WVP. It should be noted that the above interpretations are inferred from the combined SEM, XRD, wettability, and WVP results, while direct evidence regarding changes in free volume, porosity, or microstructural defects was not obtained in the present study. Therefore, further characterization is required to fully elucidate the underlying mechanism governing water vapor transport in these membranes. Studies have demonstrated that packaging films with high WVP are more suitable for fresh foods, whereas those with low WVP are more appropriate for processed foods [51].

From a food-packaging perspective, the tunable WVP achieved through DMY loading modulation may provide useful information for the material design of membranes intended for food systems with different moisture requirements. Membranes with moderate WVP values at intermediate DMY loading levels may be further evaluated for food systems requiring balanced moisture regulation, such as fresh produce, where excessive moisture accumulation and limited gas exchange are potential concerns. In contrast, the higher WVP observed at the D5 loading suggests that this formulation may be less suitable for low-moisture foods that require stronger moisture barriers, but it may still be relevant to food systems requiring higher water vapor transmission. These observations suggest that modulation of DMY loading provides a potential approach for tuning the moisture barrier properties of the nanofibrous membranes. However, the suitability of specific formulations for particular food systems should be further validated through application-oriented studies, including standardized food-simulant tests, food-storage experiments, and shelf-life assessments.

### 3.8. Mechanical Properties

**Figure 10:**
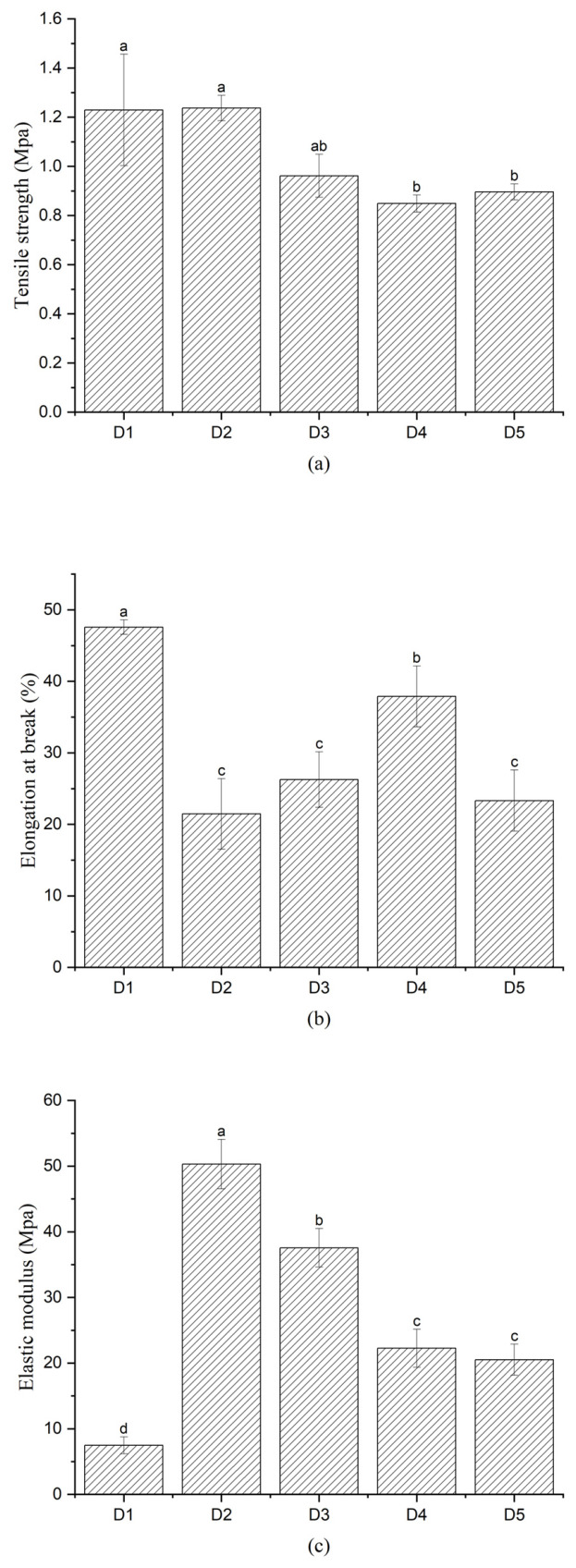
Mechanical properties of PVA/gelatin/zein nanofibers loaded with 0%, 20%, 22.5%, 25%, and 27.5% DMY: (**a**) tensile strength (TS), (**b**) elongation at break (EB) and (**c**) elastic modulus (EM). These samples were designated as D1, D2, D3, D4, and D5, respectively. Different superscripts within the same column indicate values that differ significantly (*p* < 0.05).

Mechanical properties are a core indicator for evaluating the applicability of nanofiber-based active packaging materials. Figure 10 illustrates the effects of different DMY loadings on the TS, EB, and EM of the PVA/gelatin/zein nanofibrous membranes. The results show that the introduction of DMY induced a dramatic increase in material stiffness. The EM of D2 (low DMY concentration) increased from 7.56 MPa (D1) to 50.37 MPa, an approximately 7-fold increase. This substantial increase in EM may be associated with the interactions between DMY molecules and the polymer matrix (PVA, gelatin, and zein), as suggested by the FTIR results [52]. These interactions could potentially restrict polymer chain mobility and contribute to the increased stiffness of the nanofibrous membranes. However, the exact molecular mechanism underlying this behavior was not directly investigated in the present study.

Meanwhile, a gradual decline in TS was observed with increasing DMY loading. Statistical analysis indicated that the TS values of the low-concentration samples (D2 and D3) were not significantly different from that of the control sample (D1) (*p >* 0.05), demonstrating that at low DMY loading, the material maintains macroscopic strength stability while exhibiting a substantial increase in stiffness. With increasing loading, however, the TS values of the high-concentration samples (D4 and D5) were significantly lower than that of D1 (*p* < 0.05). One possible explanation for this behavior is that DMY molecules partially altered the original intermolecular interaction network of the polymer matrix, potentially contributing to structural heterogeneity within the fibers; such structural heterogeneity may adversely affect stress distribution during mechanical deformation. At higher DMY loadings, the increased contribution of DMY-associated ordered structures indicated by XRD, together with the morphological changes observed by SEM, suggests that excessive DMY loading may affect the structural organization of the polymer matrix, thereby contributing to the observed reduction in tensile strength. Nevertheless, further investigations are required to clarify the detailed structure–property relationships governing this behavior.

Regarding the changes in EB, a pronounced nonlinear trend was observed. With the addition of DMY, the EB of D2 plummeted from 47.6% (D1) to 21.5%, corresponding to the marked increase in EM and suggesting a reduced ability of the material to undergo deformation before fracture. Notably, the EB of D4 rebounded significantly to 37.9%. One possible interpretation of this behavior is that DMY incorporation induces structural reorganization within the composite system. The combined results of XRD and mechanical testing suggest that changes in the internal organization of the nanofibrous membranes may influence their deformation behavior. However, the exact mechanism responsible for the recovery of EB at the D4 stage remains unclear and warrants further investigation [53]. The simultaneous changes observed in WVP and mechanical properties at intermediate DMY loadings suggest that DMY incorporation influences multiple structural characteristics of the membranes. However, the underlying mechanisms governing these changes remain unclear and require further investigation. In summary, the incorporation of DMY significantly altered the mechanical behavior of the nanofibrous membranes, and the resulting properties were strongly dependent on DMY loading.

From a food-packaging perspective, the observed changes in stiffness, strength, and flexibility may influence the handling, durability, and structural stability of the packaging material. The substantial increase in elastic modulus at low DMY loadings suggests improved resistance to deformation, whereas the reduction in tensile strength at high DMY loadings may limit mechanical robustness. These results indicate that DMY loading can serve as a potential parameter for tuning the mechanical performance of the nanofibrous membranes. Nevertheless, the suitability of specific formulations for particular food-packaging applications should be further assessed through application-oriented studies, including packaging performance, moisture management, and food-storage evaluations.

### 3.9. Release Analysis

**Figure 11:**
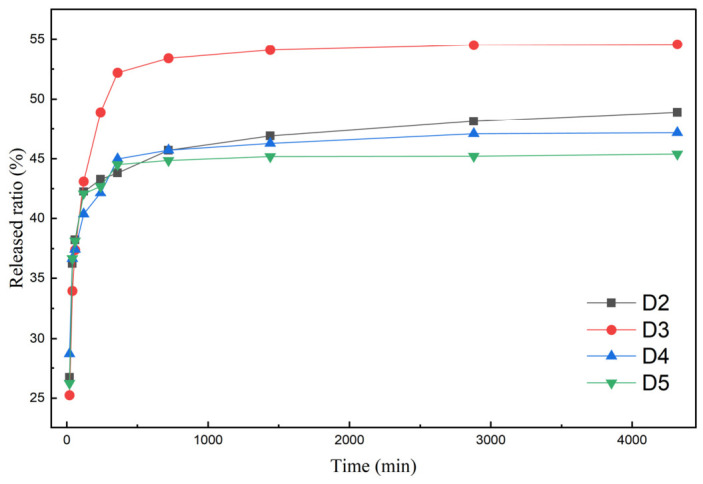
Release profiles of PVA/gelatin/zein nanofibers loaded with 20%, 22.5%, 25%, and 27.5% DMY; these samples were designated as D2, D3, D4, and D5, respectively.

The release profiles of DMY from the nanofibrous membranes are shown in Figure 11, while the corresponding kinetic fitting parameters and correlation coefficients are summarized in Table 4. All DMY release curves exhibited a typical two-phase release pattern. During the initial rapid burst release phase (within the first 500 min), the cumulative release percentage of all samples quickly exceeded 40%, which is primarily attributed to the rapid dissolution of DMY molecules distributed on and near the fiber surface. Thereafter, the release entered a slow and steady sustained release phase; after 500 min, the release rate decreased significantly, and the curves gradually approached a plateau at approximately 1000 min. To further elucidate the release mechanism, the release profiles were fitted using zero-order, first-order, and Higuchi kinetic models, and the corresponding fitting parameters are summarized in Table 4. Among the tested models, the first-order model exhibited the highest correlation coefficients (R^2^ = 0.9797–0.9998), whereas the Higuchi model showed moderate fitting quality (R^2^ = 0.6814–0.7378), and the zero-order model provided relatively poor fitting results (R^2^ = 0.2510–0.4115). These findings suggest that the release of DMY from the nanofibrous membranes is predominantly governed by concentration-dependent first-order kinetics rather than ideal zero-order release. Furthermore, the first-order release rate constant increased from D2 to D3 and subsequently decreased for D4, followed by an increase for D5, consistent with the observed variation in cumulative release. It should be noted that the superior fit of the first-order model may be partly attributed to the pronounced burst release phase, which dominates the overall release profile. Under such conditions, the first-order model mathematically captures the concentration-driven dissolution of surface-associated DMY, whereas the contribution of diffusion through the polymer matrix—while present—is not the rate-limiting step for the majority of the release process. The non-monotonic trend in the first-order rate constant may be associated with structural heterogeneity and reduced accessibility of DMY molecules within the nanofibrous matrix. Korsmeyer–Peppas fitting was not considered appropriate for the present system because the pronounced burst release behavior resulted in only a limited number of data points within the recommended fitting range (M_t_/M_∞_ < 0.6), thereby limiting the reliability of the obtained kinetic parameters.

Notably, the equilibrium cumulative release percentage of sample D3 was significantly higher than those of the other samples, with a final release percentage approaching 55%. This indicates that, at relatively low-to-moderate DMY loadings (D2–D3), a moderate increase in DMY content contributes to enhancing the ultimate DMY release amount. However, when the DMY content was further increased (samples D4 and D5), the cumulative release percentage did not continue to rise but instead fell back to the range of 45–50%.

This non-monotonic release behavior may be associated with changes in the physical state and spatial distribution of DMY within the nanofibrous matrix. At relatively low loadings, DMY was more uniformly distributed within the polymer matrix, facilitating its accessibility to the release medium. In this dispersed state, DMY molecules are readily accessible to the release medium, and the moderate increase in DMY content contributes to an increased DMY concentration gradient, thereby facilitating higher cumulative release. XRD results revealed enhanced diffraction intensity associated with DMY-rich ordered domains at higher loading levels. DSC analysis further supported that DMY remained largely dispersed within the polymer matrix without detectable crystalline melting transitions, even at elevated loadings. Taken together, these observations suggest that the increased contribution of DMY-associated ordered domains does not correspond to classical crystallization, but rather reflects changes in molecular organization and spatial distribution within the nanofibrous matrix. Within such a heterogeneous matrix, a fraction of DMY molecules may become entrapped in less accessible regions, thereby reducing the overall release efficiency. This interpretation is consistent with the SEM observations for D5, which revealed deteriorated fiber morphology and broadened diameter distribution, indicating reduced structural uniformity that may hinder DMY transport. Therefore, the release behavior of the nanofibrous membranes is influenced not only by the total DMY loading but also by its physical state, spatial distribution, and the resulting microstructural characteristics of the fibers.

It should be noted that a 20% (*v*/*v*) aqueous methanol solution was employed as the release medium, and all release experiments were conducted under sink conditions to ensure adequate solubility of DMY throughout the testing period. Although this aqueous methanol system provides a standardized and reproducible environment for comparing the release behaviors of different membrane formulations, it cannot fully mimic the complexity of real food matrices. The presence of lipids, proteins, salts, organic acids, and other food constituents may influence the migration and release behavior of DMY. Therefore, the release results obtained in this study primarily reflect the release characteristics of the nanofibrous membranes under model conditions. Further investigations using standardized food simulants and real food systems are warranted to comprehensively evaluate the release performance and practical applicability of these nanofibrous membranes in food-packaging applications.

### 3.10. Antioxidant Activity Analysis

**Figure 12:**
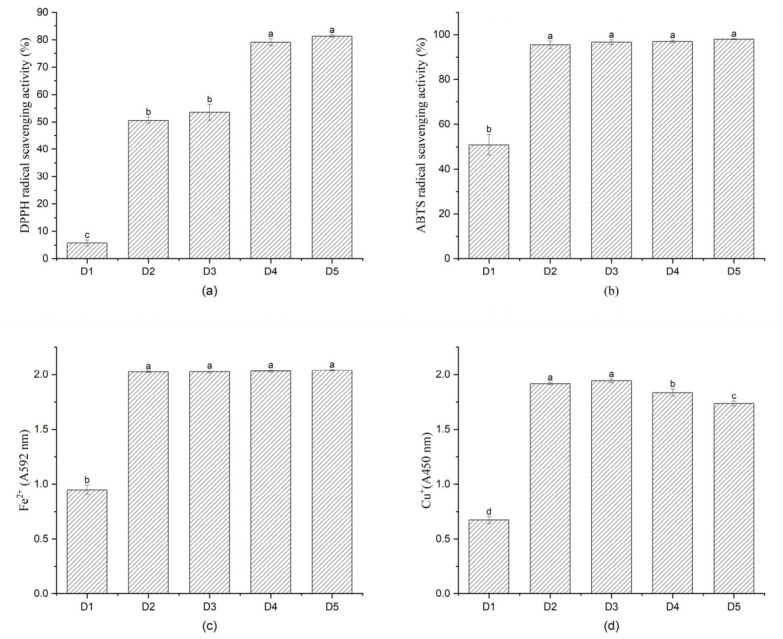
Antioxidant activities of PVA/gelatin/zein nanofibers loaded with 0%, 20%, 22.5%, 25%, and 27.5% DMY: (**a**) DPPH radical scavenging activity; (**b**) ABTS radical scavenging activity; (**c**) Fe^3+^ reducing power; (**d**) Cu^2+^ reducing power. These samples were designated as D1, D2, D3, D4, and D5, respectively. Different superscripts within the same column indicate values that differ significantly (*p* < 0.05).

Food spoilage is commonly initiated by lipid oxidation and protein degradation mediated by reactive oxygen species and free radicals. It has been demonstrated that the natural flavonoid DMY possesses excellent free radical scavenging capacity [31]. In this study, the antioxidant properties of the nanofiber membranes were evaluated by DPPH radical scavenging assay (Figure 12a), ABTS radical scavenging assay (Figure 12b), ferric reducing antioxidant power (FRAP, Figure 12c), and cupric reducing antioxidant capacity (CUPRAC, Figure 12d).

The results showed that the pristine PVA/gelatin/zein nanofibers (D1) exhibited intrinsic antioxidant activity, which may be associated with antioxidant amino acid residues and peptide sequences present in gelatin, as well as sulfur-containing amino acid residues and lutein in zein [54,55]. Notably, with increasing DMY loading, the composite nanofibers displayed a significant dose-dependent increase in the DPPH and ABTS radical scavenging rates, as well as in the reducing capacities toward Fe^3+^ and Cu^2+^.

This enhancement can be ascribed to the following two aspects. First, the DMY molecule contains multiple reactive phenolic hydroxyl groups, which can efficiently donate hydrogen atoms to neutralize free radicals, thereby terminating oxidative chain reactions.

Second, the incorporation of DMY into the nanofibrous matrix, combined with the high specific surface area of the nanofibers, may facilitate interactions between DMY and the reaction medium. The intermolecular hydrogen bonding revealed by FTIR analysis suggests potential interactions between DMY and the polymer matrix, which may contribute to the retention of DMY within the nanofibers. It should be noted that the release behavior of DMY from these nanofibrous membranes has been systematically investigated in the preceding section; the antioxidant assays reported here primarily reflect the overall radical scavenging capacity of the membranes as a whole. Further investigation is required to decouple the relative contributions of released DMY and matrix-bound DMY to the observed antioxidant activity.

### 3.11. Antibacterial Activity Analysis

**Figure 13:**
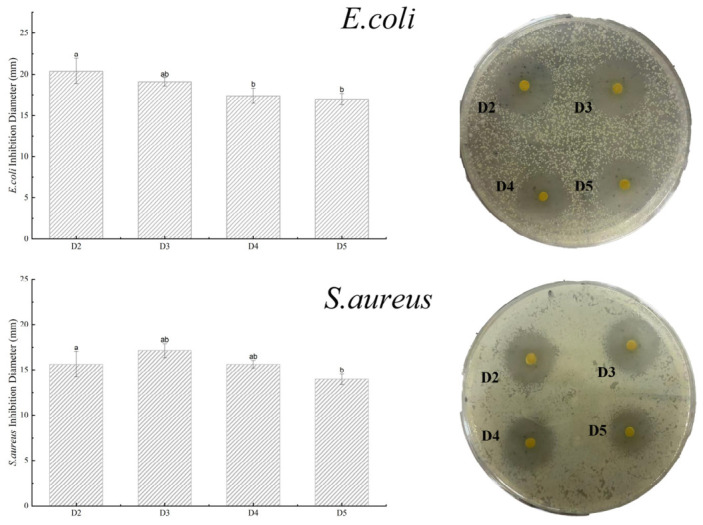
Antibacterial activity of PVA/gelatin/zein nanofibers loaded with 0%, 20%, 22.5%, 25%, and 27.5% DMY, these samples were designated as D1, D2, D3, D4, and D5, respectively. Different superscripts within the same column indicate values that differ significantly (*p* < 0.05).

As shown in Figure 13, the DMY-loaded nanofibers produced clear inhibition zones against both *Escherichia coli* (*E. coli*) and *Staphylococcus aureus* (*S. aureus*), indicating that the DMY-loaded nanofibers exhibited antibacterial activity against the tested representative Gram-negative and Gram-positive bacterial strains. Notably, the antibacterial efficacy did not increase linearly with increasing DMY content; instead, the inhibition zone diameter decreased slightly when the concentration reached 27.5%.

This non-monotonic structure–activity relationship can be understood in the context of the structural evolution of the nanofibrous membranes with increasing DMY loading, as revealed by the combined XRD, DSC, TGA, SEM, and in vitro release results. At low to moderate DMY loadings, DSC analysis further supported that DMY remained largely dispersed within the polymer matrix without detectable crystalline melting transitions. In this dispersed state, DMY molecules are readily accessible to the agar medium upon contact, allowing effective diffusion and the establishment of a locally high antibacterial concentration around the fibers. At the highest DMY loading (27.5%), XRD analysis indicated an enhanced contribution of DMY-associated ordered structures, while TGA results revealed changes in the thermal behavior of the system. SEM observations further revealed deteriorated fiber morphology and broadened diameter distribution, indicating reduced structural uniformity. As discussed in the in vitro release section, such structural heterogeneity may result in a fraction of DMY molecules becoming entrapped in less accessible regions within the fiber matrix, thereby limiting their diffusion into the surrounding agar medium. Consequently, the reduced local concentration of bioavailable DMY around the fibers may account for the slight decrease in inhibition zone diameter at the D5 level.

Although the present system did not show clear evidence of classical DMY crystallization, the study of Xu et al. [41] highlights the importance of the physical state and molecular organization of DMY in determining its bioavailability and bioactivity. In the present PVA/gelatin/zein nanofiber system, the polymer matrix appears capable of maintaining DMY in a relatively well-dispersed state at low to moderate loadings, thereby preserving its antibacterial activity. However, at excessively high loadings, the structural heterogeneity induced by excessive DMY appears to reduce the accessibility of some DMY molecules, which may contribute to the slight decline in antibacterial performance. Therefore, maintaining an appropriate DMY loading level and a structurally homogeneous distribution within the polymer matrix may be beneficial for maximizing the antibacterial activity of the nanofibrous membranes.

From a food-packaging perspective, the antibacterial activity of the DMY-loaded nanofibrous membranes may provide useful information for future active-packaging studies. However, because the present antibacterial evaluation was limited to *E. coli* and *S. aureus*, these results should be regarded as preliminary evidence of antibacterial activity against the tested bacteria rather than broad antimicrobial or antifungal performance. Although the inhibition zone assay demonstrated that the DMY-loaded nanofibrous membranes possessed promising antibacterial activity against both *E. coli* and *S. aureus*, it should be noted that the agar diffusion method is influenced by the diffusion behavior of active compounds within the culture medium and therefore provides only a preliminary assessment of antibacterial performance. More comprehensive microbiological evaluations, including minimum inhibitory concentration (MIC), minimum bactericidal concentration (MBC), bacterial viability assays, and time–kill studies, are required to further elucidate the antibacterial efficacy and mechanism of the membranes. These investigations are required before practical food-preservation performance can be established.

## 4. Conclusions

In this study, poly(vinyl alcohol) (PVA)/gelatin/zein nanofibers loaded with dihydromyricetin (DMY) were successfully fabricated via electroblowing spinning. FTIR spectroscopy suggested the possible formation of hydrogen-bonding interactions between DMY and the polymer matrix, while XRD and DSC analyses indicated that the physical state and distribution of DMY gradually changed with increasing loading. These structural changes were closely associated with variations in fiber morphology, hydrophilicity, mechanical properties, and release behavior.

The nanofiber encapsulation system provided a controlled and sustained release profile for DMY. Among the tested formulations, the membrane with 22.5% DMY showed the highest cumulative release efficiency of nearly 55%, likely due to a more favorable distribution of DMY within the polymer matrix. In contrast, excessive DMY loading led to structural heterogeneity and reduced release efficiency, highlighting the importance of optimizing the loading level and physical state of DMY.

The DMY-loaded nanofibrous membranes exhibited DMY-dependent antioxidant activity and antibacterial activity against E. coli and S. aureus. Based on their hydrophilic nature, tunable water vapor permeability, controlled DMY release, antioxidant activity, and antibacterial performance against the tested bacteria, these findings may provide useful information for future active food-packaging studies involving food systems with different moisture requirements. However, because ^1^H NMR analysis, biocompatibility and migration tests, standardized food-simulant studies, antifungal assays, and real-food storage experiments were not conducted in the present study, the suitability of these membranes for specific food products and practical food-packaging applications requires further validation. Therefore, the present work should be regarded as a preliminary material-fabrication and characterization study rather than a complete validation for practical food-packaging applications.

## Figures and Tables

**Table 1:** Gradient elution program for liquid chromatography.

Time (min)	Flow Rate (mL/min)	A%	B%
0	0.5	98	2
1	0.5	98	2
10	0.5	70	30
20	0.5	0	100
25	0.5	0	100
26	0.5	98	2
36	0.5	98	2

**Table 2:** Power-law index (n) and consistency index (K) of PVA/gelatin/zein composite solutions loaded with 0%, 20%, 22.5%, 25%, and 27.5% DMY; these samples were designated as D1, D2, D3, D4, and D5, respectively.

Sample	n	K (Pa·s^n^)
D1 (0%)	0.91 ± 0.02 ^a^	0.41 ± 0.06 ^a^
D2 (20%)	0.92 ± 0.01 ^ab^	0.32 ± 0.02 ^b^
D3 (22.5%)	0.90 ± 0.01 ^b^	0.22 ± 0.03 ^b^
D4 (25%)	0.89 ± 0.01 ^b^	0.46 ± 0.07 ^a^
D5 (27.5%)	0.88 ± 0.01 ^ab^	0.57 ± 0.05 ^a^

Different superscripts within the same column indicate values that differ significantly (*p* < 0.05).

**Table 3:** TGA data of PVA/gelatin/zein nanofibers loaded with 0%, 20%, 22.5%, 25%, and 27.5% DMY; these samples were designated as D1, D2, D3, D4, and D5, respectively.

	TGA						
Sample	Peak1 (°C)	Weight Loss (%)	Peak2 (°C)	Weight Loss (%)	Peak3 (°C)	Weight Loss (%)	Residue at 604 °C (%)
D1 (0%)	54.71	4.32	197.37	8.52	312.94	55.93	18.13
D2 (20%)	53.60	5.44	195.77	7.03	311.06	49.88	25.52
D3 (22.5%)	57.43	5.03	193.24	6.64	312.92	50.66	24.12
D4 (25%)	59.54	4.41	192.69	6.45	311.64	49.60	25.23
D5 (27.5%)	57.66	4.46	190.27	5.93	310.95	49.67	25.89

**Table 4:** Kinetic fitting parameters of DMY release from nanofibrous membranes based on zero-order, first-order, and Higuchi models. R^2^ represents the coefficient of determination for each model, and k denotes the apparent release rate constant of the first-order model.

Sample	Zero-Order R^2^	First-Order R^2^	Higuchi R^2^	First-Order k
D2	0.41149	0.9961	0.7071	0.0725
D3	0.3610	0.9970	0.7378	0.0830
D4	0.3946	0.9797	0.6971	0.0486
D5	0.2510	0.9998	0.6814	0.0807

## Data Availability

The original contributions presented in the study are included in the article; further inquiries can be directed to the corresponding author.
